# Expression of Concern: miR-517a is upregulated in glioma and promotes glioma tumorigenesis in vitro and in vivo

**DOI:** 10.1042/BSR-2018-1196_EOC

**Published:** 2022-10-26

**Keywords:** glioma, invasion, migration, miR-517a, proliferation

The Editorial Office has been made aware of potential issues surrounding the scientific validity of this paper, hence has issued an expression of concern to notify readers whilst the Editorial Office investigates. It is noted that similarities exist between the Figure 6A tumour images and Figure 4 from Sun et al. 2016 (DOI: 10.1007/s13277-016-5444-9) and Figure 3A from Kong et al. 2015 (DOI: 10.1186/s12943-015-0355-8). Similarities also exist between the cell images from Figure 6D (rotated 90 degrees) and Figure 5D from Han et al. 2015 (10.1038/cddis.2015.30), and the western blots from Figure 5B and those from Figure 6C of the same paper (Han et al. 2015).

